# DNA analysis of traded shark fins and mobulid gill plates reveals a high proportion of species of conservation concern

**DOI:** 10.1038/s41598-017-10123-5

**Published:** 2017-08-25

**Authors:** Dirk Steinke, Andrea M. Bernard, Rebekah L. Horn, Paul Hilton, Robert Hanner, Mahmood S. Shivji

**Affiliations:** 1 Centre for Biodiversity Genomics, University of Guelph, 50 Stone Road East, Guelph, Ontario, N1G 2W1 Canada; 2 Save Our Seas Shark Research Center USA and Department of Biological Sciences, Nova Southeastern University, 8000 North Ocean Drive, Dania Beach, FL 33004 USA; 3 Guy Harvey Research Institute and Department of Biological Sciences, Nova Southeastern University, 8000 North Ocean Drive, Dania Beach, FL 33004 USA; 4 Paul Hilton Photography, Hong Kong, China; 5 Biodiversity Institute of Ontario, University of Guelph, 50 Stone Road East, Guelph, Ontario, N1G 2W1 Canada

## Abstract

Continuously increasing demand for plant and animal products causes unsustainable depletion of biological resources. It is estimated that one-quarter of sharks and rays are threatened worldwide and although the global fin trade is widely recognized as a major driver, demand for meat, liver oil, and gill plates also represents a significant threat. This study used DNA barcoding and 16 S rRNA sequencing as a method to identify shark and ray species from dried fins and gill plates, obtained in Canada, China, and Sri Lanka. 129 fins and gill plates were analysed and searches on BOLD produced matches to 20 species of sharks and five species of rays or – in two cases – to a species pair. Twelve of the species found are listed or have been approved for listing in 2017 in the appendices of the Convention on International Trade in Endangered Species of Fauna and Flora (CITES), including the whale shark (*Rhincodon typus*), which was surprisingly found among both shark fin and gill plate samples. More than half of identified species fall under the IUCN Red List categories ‘Endangered’ and ‘Vulnerable’, raising further concerns about the impacts of this trade on the sustainability of these low productivity species.

## Introduction

Shark and ray (Elasmobranchii) fisheries have expanded in size and number around the world since the mid-1980s, with many species directly targeted or taken as by-catch in fisheries targeting teleosts. Elasmobranch meat is an important source of protein in many coastal communities. However, high demand for their fins to make soup, a celebratory dish in some Asian cuisines, and more recently, for the prebranchial appendages or gill plates (rakers) of mobulid rays (Family Mobulidae) for medicinal purposes is driving the unsustainable exploitation of these vulnerable fishes^1–4^.

Sharks sourced for fins and mobulid rays for gill plates come from various sources such as legal fisheries, by-catch, and illegal, unreported and unregulated (IUU) fisheries. The number of sharks and rays caught as undeclared by-catch and in IUU fisheries likely far outweighs those from legal fisheries. An analysis of trade data from Asian markets^1^ estimated that 26–73 million sharks per year are caught for the fin trade alone, a value 3–4 times higher than the United Nations Food and Agriculture Organization (FAO) estimates for this trade segment. The estimated annual global volume of the mobulid gill plate trade in 2011 was 61,000 kg of dried gills, worth US$11.3 million and representing about 5000 rays^5^. More recent estimates based on trader surveys in southern China place the number of rays contributing to the gill plate trade at 130 000 animals in 2013^6^. Population declines of 56–86% for mobulids have been reported over the past 6–8 years in key range countries such as Indonesia and Mozambique resulting from increased exploitation and habitat destruction^5, 7–10^.

Life history characteristics such as low fecundity, slow growth, long lifespan, and late maturity result in a low rate of population growth in many elasmobranch species, which makes them more vulnerable to fishing pressure than most traditional target teleost species^11, 12^. An IUCN study^13^ found that as a result 32% of all pelagic shark and ray species are now at risk of extinction. These concerns, in the context of continued demand and trade in shark fins and mobulid gill plates, has resulted in eight shark species (i.e., whale, basking, white, porbeagle, oceanic whitetip, scalloped hammerhead, smooth hammerhead and great hammerhead sharks) and two mobulid ray species (giant manta and reef manta rays; genus *Manta;* hereafter “manta rays”) being listed on Appendix II of the Convention on International Trade in Endangered Species of Fauna and Flora (CITES). Furthermore, in a recent development, all the nine known mobulid species in the genus *Mobula* (devil rays) have now also been approved for CITES Appendix II listing starting on April 4, 2017, because of strong concerns about their high extinction risk in the face of even low fishing mortality^3, 6, 14^. The CITES listing requires international trade in these species to be highly regulated, including an assessment by the exporting country that the trade will not be detrimental to wild populations of the species^15^. Given a paucity of trade records, in part because of species identification difficulties, the relative contribution of each of the nine devil ray species to the global gill plate trade requires further investigation^4^.

An accurate picture of the population status and fishery sustainability of elasmobranchs worldwide requires major improvements to species catch data records with one of the limiting factors being the problem of accurate identification of morphologically similar species and processed body parts. Dried fins and gill plates typically lack clear diagnostic features, making morphological species identification difficult, or in some cases impossible. Identification is further complicated by the use of a plethora of market categories that do not necessarily correspond to taxonomic names^16, 17^.

Over the past few decades, molecular techniques have been proposed for elasmobranch species identification, particularly in instances when traditional taxonomic methods fail^17–22^. DNA barcoding has been established as a powerful tool for fish species identification^23, 24^, and several studies have successfully barcoded sharks and rays^25, 26^, as well as processed material^4, 27–31^. As of February 2017, some 670 of the known 1200 elasmobranch species have been barcoded. In our study, DNA identification via mitochondrial COI barcoding and 16 S ribosomal gene sequencing was used to identify dried shark fins obtained at retailers in Vancouver, Canada, and mobulid gill plates obtained from retailers in China and from fisheries in Sri-Lanka.

## Results

**Figure 1:**
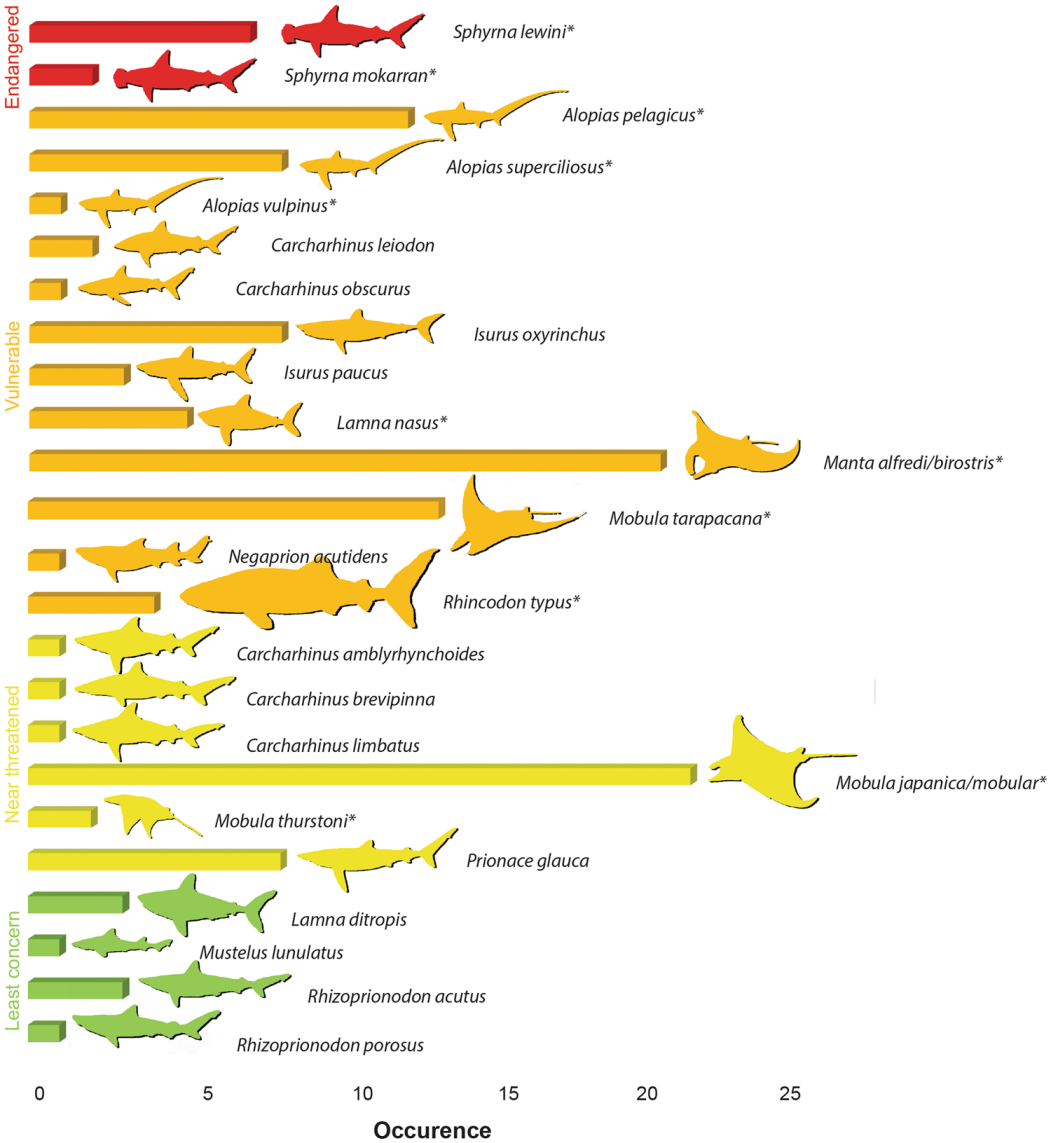
Species identifications for 134 dried shark fin and Mobulidae ray gill plate samples. Bar length represents abundance; colour and order (Y-axis) correspond to IUCN Red List status. Species currently listed or that will be listed in CITES appendices in 2017 are marked with CITES logo.

Good quality COI barcode sequences were obtained for all 71 dried shark fin samples and 53 of the 58 dried gill plate samples, with sequence lengths varying between 93 and 664 bp (average length 508 bp). For the five remaining dried gill plates good quality sequences were obtained via 16 S rRNA sequencing; these 16 S sequences varied in length between 505–514 bp (average length 511 bp). Most barcode searches on BOLD produced clear matches allowing for confident assignment of species with > 99% similarity to database records. However, for the gill plates, 21 barcode query sequences had a > 99% match to BOLD sequences from both *Mobula japonica* and *Mobula mobular*. Additionally, another 20 gill plate barcodes matched both *Manta alfredi* and *Manta birostris*. Using NCBI’s BLAST tool, all 16 S rRNA sequences were identified as belonging to *Mobula tarapacana* (GenBank Accession #KM364986; 99–100% sequence identity). Across the entire study, 20 species of sharks and at least 5 species of mobulid rays (assuming *Mobula japonica* and *Mobula mobular* are not different species^32^) were identified in the traded products. The number of specimens per species ranged from one (eight species) to 21 (*Mobula japanica/mobular* complex). Five of the species found are listed in the current CITES appendices (Fig. 1), and six species (three *Mobula* and three *Alopias* species) have been approved for listing on CITES Appendix II starting in April and October, 2017, respectively. The COI sequences revealed both fin and gill plate samples from the whale shark (*Rhincodon typus*), currently a CITES Appendix II listed species, in the retail markets. More than half of the species found (14) fall under the IUCN RedList threatened categories Endangered (2) and Vulnerable (12). Six more species are categorized as Near Threatened, and four are in the lowest risk category (Fig. 1).

## Discussion

In this study, we identified 129 commercial samples using the BOLD “species-level” identification tool to query sample barcodes against the BOLD reference database or using NCBI’s BLAST tool, which provided a sequence similarity value greater than 99% in all cases, despite considerable length differences of the query sequences. Given the incompleteness of the reference database for the world’s elasmobranchs it seems likely that not every fin or gill plate confiscated or collected can be presently identified by DNA barcoding. However, earlier efforts of the global initiative to barcode all fishes, FishBOL largely focused on species of commercial interest^33^. In the case of elasmobranchs sampling is frequently opportunistic, which means samples are often sourced through trade and/or commercial fish landings as body parts, making correct species determinations by morphology more unlikely. Even if identification fails at this point in time, barcodes obtained in studies such as this are available for subsequent matching as the reference database expands.

Poor preservation conditions as well as various processing methods, e.g. shark fins and gill plates are usually air dried in the open until they are shipped to the point of sale, let tissue and DNA slowly deteriorate which in turn can have a profound effect on sequencing success. Shorter reads are often the result of DNA degradation but it has been shown that such mini-barcodes still show sufficient sequence variation and divergence to provide the same information as a full length barcode sequence^34–36^. A number of short sequences obtained from shark fins for this study (29) ranged in length from 93 bp to 184 bp. Despite this, all of them were unambiguously matched to single shark species reference sequences on BOLD, demonstrating that each fragment contained enough variation for a successful identification.

Barcode sequences obtained for 41 gill plate samples could not be unambiguously matched to a single species on BOLD. Some results (21 samples) with similarity values > 99% pointed to both *Mobula japanica* and *Mobula mobular* (currently listed as near threatened on the IUCN RedList). In fact all available sequences for these species on BOLD fall into the same BIN cluster^37^ which indicates that there is insufficient variation to separate both species using COI barcodes. However, a recent phylogenetic study on manta and devil rays used a combination of genetic (both nuclear and mitochondrial DNA loci) and morphological data and its findings challenge the notion that *Mobula japanica* or *Mobula mobular* are two separate species^32^.

In contrast *Manta alfredi* and *Manta birostris* are thought to be sibling species that have speciated relatively recently^38^. In addition, Walter *et al*.^39^ reported hybrids between the two species indicating incomplete lineage sorting. Consequently, DNA barcodes of both species fall into the same cluster indicating that COI is insufficient to delineate them despite some weak phylogenetic structure^25^. In a study of 188 market-derived gill plates from southern China, Zeng *et al*.^4^ also reported the occurrence gills from the genus *Manta* (11.2% of samples). However, they also were unable to distinguish between the two known *Manta* species based on COI barcodes, nor mitochondrial NADH2 gene sequences, and chose to assign their products to *Manta birostris*. It remains possible that some portion of the gill samples in both our study and Zeng *et al*.^4^ originated from *Manta alfredi*.

Continuously increasing demand for plant and animal products causes unsustainable depletion of biological resources^40, 41^. However, we are only now just beginning to understand the enormous scale of trade in aquatic organisms, such as the live food fish trade^42^, or the dried product trade in shark fins^1^, and manta and devil ray gill plates^4, 6, 12^.

Our market samples comprised 20 species of sharks and at least 5 species of mobulid rays. Twelve of these species (seven sharks and five rays) are or will soon be (in 2017) listed on Appendix II of CITES (Fig. 1). We note that most of our market samples were obtained in 2011 and 2012 and thus predated, with the exception of the whale shark (CITES listed in 2003), the 2014 and onwards inclusion of these species in CITES listings. However, the confirmed occurrence of these species’ body parts in recent trade suggests ongoing market demands, and it’s probable that trade in these species, even post-CITES listing, is occurring to various extents. Also of note is the finding of both fin and gill plate products from the widely protected whale shark, raising the issue of whether whale shark gills are now also entering trade because of a specific demand, or their occurrence is simply an attempt at surreptitious substitution for mobulid gills.

In addition to the CITES enforcement ramifications of our findings, of particular concern is that 56% of species found are IUCN Red List categorized as Endangered and Vulnerable, with 80% falling into the Red List categories of Endangered, Vulnerable or Near Threatened (Fig. 1). Under CITES Appendix II regulations, any trade of listed shark or ray species products requires proof that nations have methods in place that allows assessments to ensure the proposed trade is sustainable and not detrimental to any wild populations. Given that sharks and rays are typically characterized by low fecundity, late maturity, and a long gestation periods^11, 12^, the finding that 71% of the all fins and gill plates sampled came from species of high conservation concern suggests that this segment of the global fisheries is anything but sustainable, and urgently requires an extensive conservation management response.

The DNA analysis of our trade-obtained samples, which included Hong Kong and Sri Lanka sources, adds to the findings of Zeng *et al*.^4^ who reported gill plates of the mobulid species *Mobula japanica*, *Mobula tarapacana*, *Mobula thurstoni* and *Mobula kuhlii* in markets of the Guangdong Province and Guangxi Zhuang Autonomous Region of southern Mainland China. We did not find gills originating from *M*. *kuhlii* (IUCN Data Deficient listing), an apparently uncommon species^43^. However, the proportion of gill plates from *M*. *tarapacana* (IUCN Vulnerable listing) made up about a quarter of our gill plate samples (22%; cf. 14.4% found by Zeng *et al*.^4^); the higher prices of gills from this species compared to other mobulids in the trade^4, 6^ may reflect a market preference for this species because of its longer gill filaments^4^, and points to the need for increased information to understand impacts of the trade on this species. Similarly, the IUCN Vulnerable listed thresher sharks (*Alopias* spp.) comprised 30% of all fins tested. The high frequency of these species suggests that they are neither by-catch nor imported from small-scale fisheries, but instead harvested through large-scale fisheries. This was also documented by other barcoding studies with a focus on shark products^29, 30^.

Our study confirmed that DNA identification of dried shark fins and mobulid gill plates via mitochondrial COI barcoding and 16 S ribosomal gene sequencing represents a powerful market surveillance tool. While shark conservation and management policies are primarily focused on the development of fisheries quotas and marine protected areas^44^, this work demonstrates the importance of market surveillance as a conservation countermeasure that would benefit from large-scale long-term monitoring. The results also raise further concerns about the impacts of this trade on the sustainability of these low productivity species demanding swift and extensive conservation management responses.

## Methods

A total of 129 samples were purchased or obtained cost-free from various retailers (71 dried shark fins from Vancouver, 54 gill plates from Hong Kong and mainland China), and fisheries in Sri-Lanka (four gill plates) over the years 2011 and 2012. After acquisition, specimen tissues were subsampled for analysis. All shark fin specimen provenance data, including the market name, were recorded locally. A detailed overview of the samples is given in the public dataset DS-SHMA (dx.doi.org/10.5883/DS-SHMA) on the Barcode of Life Datasystems (BOLD)^45^. All sequences in this study have also been deposited in GenBank. Accession numbers are available through the BOLD dataset.

Genomic DNA extractions were performed by using either a Glass Fibre Plate DNA Extraction (AcroPrep) method^46^ or by use of DNeasy Blood & Tissue Kits (QIAGEN). For all dried shark fins, the 652 bp barcode region of COI was subsequently amplified under the following thermal conditions: 2 min at 95 °C; 35 cycles of 0.5 min at 94 °C, 0.5 min at 52 °C, and 1 min at 72 °C; 10 min at 72 °C; held at 4 °C. The 12.5 µl PCR reaction mixes included 6.25 µl of 10% trehalose, 2.00 µl of ultrapure water, 1.25 µl 10X PCR buffer [200 mM Tris-HCl (pH 8.4), 500 mM KCl], 0.625 µl MgCl2 (50 mM), 0.125 µl of each primer cocktail (0.01 mM, using primer cocktails C_FishF1t1 and C_FishR1t1 or C_VF1LFt1 and C_VR1LRt1, alternatively^47^, 0.062 µl of each dNTP (10 mM), 0.060 µl of Platinum® Taq Polymerase (Invitrogen), and 2.0 µl of DNA template. PCR amplicons were visualized on a 1.2% agarose gel E-Gel® (Invitrogen) and bidirectionally sequenced using sequencing primers M13F or M13R^48^ and the BigDye® Terminator v.3.1 Cycle Sequencing Kit (Applied Biosystems, Inc.) on an ABI 3730xl capillary sequencer following manufacturer’s instructions.

For gill plates, this same COI barcode region was targeted for amplification using the primer suite outlined in Ward *et al*.^23^ (FishF1, FishF2, FishR1, and FishR2) following a similar thermal profile: 15 min at 95 °C; 35 cycles of 1 min at 94 °C, 1 min at 50 °C, and 2 min at 72 °C; 20 min at 72 °C. The 50 µl PCR reaction mixes consisted of 33.3 µl of OmniSolv® Water, Gradient Grade, 5 µl of 10X CoralLoad PCR buffer (15 mM MgCl_2_), 1.25 µl of each primer (10 µM), 4 µl of each dNTP (1.25 mM of each dNTP), 0.2 µl of HotStar Taq DNA Polymerase (QIAGEN), and 1 µl of DNA template. PCR amplicons were sequenced bi-directionally using a combination of Ward *et al*.’s COI primer suite^23^, and using the BigDye® Terminator v.3.1 Cycle Sequencing Kit (Applied Biosystems, Inc.) on an ABI 3130 capillary sequencer.

Where COI amplification and DNA sequencing of gill plate samples was unsuccessful (N = 5), the mitochondrial 16 S rRNA gene was targeted for sequencing to achieve molecular species identification. Using an identical reaction protocol, thermal profile, and sequencing conditions as outlined above for gill plate COI sequencing, the primer suite 16sarl-L and 16sbr-H^49^, was used to amplify and sequence an ~ 500 bp region of the 16 s rRNA gene.

Bi-directional sequences were assembled and edited using CodonCode Aligner software (CodonCode Corporation, USA) or Geneious v6.1.8 (Biomatters Ltd., Auckland)^50^. We analyzed the DNA barcode sequences derived from the fin and gill plate samples with the “species”-level identification function of the BOLD ID Engine (version July 2016). A top species match was identified with a sequence similarity of at least 99%. Species-level identification of gill plates sequenced at the 16 S rRNA gene was performed using the Basic Local Alignment Search Tool (BLAST) algorithm of the National Center for Biotechnology Information (NCBI). A top species match was identified with a sequence similarity of at least 99%.
